# Computed Tomographic Findings in Dogs with Hepatic Bacterial Parenchymal Infection and Abscessation

**DOI:** 10.3390/ani14233399

**Published:** 2024-11-25

**Authors:** Luis Maté de Haro, Andrea Vila, Andrea Di Bella, Claudia Mallol, Carlo Anselmi, Jose-Daniel Barreiro-Vazquez, Danica Pollard, Raquel Salgüero, Ella Fitzgerald, Beatriz Moreno-Aguado

**Affiliations:** 1 Southern Counties Veterinary Specialists, Independent Vetcare (IVC) Evidensia, Forest Corner Farm, Hangersley, Ringwood BH24 3JW, UK; 2 Department of Clinical Sciences and Services, The Royal Veterinary College, University of London, Hertfordshire AL9 7TA, UK; 3 Anderson Moores Veterinary Specialists, The Granary, Bunstead Barns, Poles Ln, Winchester SO21 2LL, UK; 4 Blaise Veterinary Referral Hospital, Independent Vetcare (IVC) Evidensia, 1601 Bristol Road South, Longbridge, Birmingham B45 9UA, UK; 5 Departamento de Anatomía, Produción Animal e Ciencias Clínicas Veterinarias, Facultade de Veterinaria, Universidade de Santiago de Compostela, Hospital Veterinario Universitario Rof Codina, Estrada da Granxa, 51, 27002 Lugo, Spain; 6 Independent Researcher, The Rodhams, Rodham Road, Wisbech PE14 9NU, UK; 7 Hospital Veterinario Veterios, C. de Arrastaria, 23, San Blas-Canillejas, 28022 Madrid, Spain

**Keywords:** computed tomography, canine, bacterial liver infection, liver abscess

## Abstract

Bacterial liver parenchymal infections in dogs are rarely documented, and their imaging characteristics are scarce in the veterinary literature, especially in Computed Tomography (CT). This retrospective multicentric study aimed to describe the CT characteristics of parenchymal bacterial liver infection and abscessation in dogs and compare them with the human literature. Twenty dogs met the inclusion criteria. All dogs, except one, showed discrete hepatic lesions consistent with pyogenic liver abscess (19/20). A single case showed diffuse liver changes, which was diagnosed with granulomatous bacterial hepatitis (1/20). Multifocal lesions were associated with the presence of abdominal pain (*p* = 0.023). CT characteristics of pyogenic liver abscesses in our study resemble those described in the human literature, with multifocal (14/19) or single (5/19), round or ovoid (19/19), hypoattenuating hepatic lesions, which are better visualised in post-contrast images. Pyogenic liver abscesses can also show features such as the “cluster sign” (8/19), transient arterial segmental enhancement (6/10), rim enhancement (6/19), and intralesional gas (4/19). Additional CT findings, such as local lymphadenomegaly (18/20), peritoneal fat stranding (14/20), and peritoneal fluid (13/20), are also commonly observed.

## Simple Summary

Infections of the liver in dogs are rare, and their Computed Tomography (CT) characteristics have not been extensively described. In this study, we compare the CT characteristics of a population of 20 dogs diagnosed with liver infections with the reported features of this disease in human patients. All the patients but one showed discrete lesions in the liver on CT that resembled pyogenic abscesses in humans. In one case, diffuse liver changes were observed. Reported common features of liver infections in humans, such as the “cluster sign”, transient arterial segmental enhancement, rim enhancement, and intralesional gas, were observed in our patients. Features of pyogenic liver abscesses in CT in this population of dogs resemble those described for human patients.

## 1. Introduction

Bacterial infection is a potential cause of canine hepatitis; however, the presence of bacteria within the liver is rarely documented [1]. The World Small Animal Veterinary Association, Liver Standardisation Group, recognises three categories of bacterial liver infections: acute bacterial hepatitis, bacterial granulomatous liver disease, and pyogenic liver abscess [2]. These entities are similarly reported in humans [3,4]. Multiple routes of bacterial infection have been described in dogs, including the biliary tract, haematogenous spread, penetrating wounds, iatrogenic, closed traumatism, or neoplasia [5,6]. Multiple bacterial agents have been associated with canine hepatitis and abscessation, including enteric bacteria (*Escherichia coli*, *Enterococcus* spp., *Bacteroides* spp., *Streptococcus* spp., *Clostridium* spp., *Salmonella* and *Helicobacter canis*) and non-enteric bacteria (*Leptospira* spp., *Bartonella* spp., *Clostridium piliform*, and *Mycobacterium* spp.) [1,7].

In humans, Computed Tomography (CT) is particularly useful in diagnosing focal and multifocal infectious hepatic processes, and it is considered the main imaging modality for the diagnosis of liver abscesses with a sensitivity of up to 97% versus a sensitivity of 85% in ultrasound [8,9,10,11,12]. The CT features of human pyogenic liver abscesses include well-defined, low-attenuation, round masses with a peripheral contrast-enhancing rim, occasionally containing gas in up to 20% of the cases [3]. Other more specific CT characteristics have also been described, such as the “cluster sign” (multiple small, low-attenuation lesions aggregated into a single larger cavity) [3,13], transient segmental or wedge-shaped arterial hepatic enhancement [9,14] or “double target sign” (two concentric rings of varying attenuations surrounding a central hypoattenuating fluid-filled area) [15]. The imaging findings of acute hepatitis in humans are nonspecific and limited to hepatomegaly, gallbladder wall oedema, and periportal oedema [10]. Bacterial granulomatous diseases show different CT characteristics in humans depending on the infectious agent, ranging from multifocal, micro- to macronodular, hypo- or isoattenuating lesions with variable post-contrast-enhancing patterns [3].

Multiple studies describe canine bacterial hepatic abscesses in ultrasound with a typical appearance of round to oval or irregular, poorly echogenic lesions, often with central cavitation [6,16,17,18,19,20]. Intralesional gas, distal acoustic enhancement artefact, abdominal effusion, regional lymphadenopathy, and hyperechoic perihepatic fat have also been identified in dogs with hepatic abscesses in ultrasound [17]. There is limited information regarding the CT appearance of liver abscess in dogs, which includes well-defined hypoattenuating liver mass-lesions with a contrast-enhancing wall, liver emphysema, and multiple hepatic nodules, with additional findings such as peritoneal effusion, abdominal lymphadenomegaly, and pneumoperitoneum [5,20,21,22,23,24,25]. In two previous studies, CT identified liver abscesses that were missed sonographically [5,20]. Diagnostic imaging findings in acute (bacterial) hepatitis in dogs are nonspecific, being limited to ultrasonographic descriptions of hepatomegaly and diffuse decreased echogenicity [26,27], and have not been described yet in CT. The CT findings of granulomatous bacterial liver disease in dogs are limited to a single case report of *Mycobacterium tuberculosis* with multiple rounded, well-defined, hypoattenuating, and non-contrast-enhancing nodules visible throughout the liver parenchyma [28].

In this study, the authors aim to describe the CT findings of liver bacterial infections in dogs and compare them to those described in the human literature.

## 2. Materials and Methods

### 2.1. Selection and Description of Subjects

In this retrospective, descriptive, multicentre study, the medical records of dogs presented to seven European veterinary referral hospitals (Southern Counties Veterinary Specialists, Anderson Moores Veterinary Specialists, Pride Veterinary Centre, Hospital Veterinario Puchol, Hospital Veterinario Universitario Rof Codina, Royal Veterinary College, and Dick White Referrals) were reviewed to identify dogs with a diagnosis of hepatic infection. Inclusion criteria included: (a) complete abdominal CT with pre- and post-contrast series, and (b) confirmation of hepatic parenchymal infection with (1) “histology with evidence of bacteria’’ OR ‘’histology without evidence of bacteria and positive bacterial culture” OR “histology without evidence of bacteria and a positive FISH (fluorescence in situ hybridisation) or PCR for bacteria”, or (2) “cytology with evidence of bacteria’’ OR “cytology without evidence of bacteria and positive bacterial culture”. Patients were excluded if the CT study was incomplete (i.e., did not include unenhanced or enhanced series), if the samples did not achieve confirmation of the presence of bacterial liver infection, or if there was the presence of concurrent neoplastic cells in cytology/histopathology.

Information collected from the medical records included signalment (age, breed, sex, and neuter status), clinical history, clinical examination, biochemistry and completed blood count (CBC), histopathology/cytology reports, and bacterial culture.

### 2.2. Data Recording and Analysis

The CT studies were reviewed individually, followed by a consensus by an ECVDI-boarded veterinary radiologist and a second-year resident in veterinary diagnostic imaging. The readers were aware of the presence of liver infection but not the location, clinical history, or final aetiology. The images were displayed using a soft tissue window (window level 50 Hounsfield Units, or HU, window width 350 HU) for the medium-frequency reconstruction algorithm and a bone window (window level 300 HU, window width 1500 HU) for the high-frequency reconstruction algorithm. A free, open-source DICOM viewer software was used to review the images (Horos, version 3.0, Horosproject.org, Nimble Co. LLC, d/b/a Purview, Annapolis, MD, USA).

**Figure 1:**
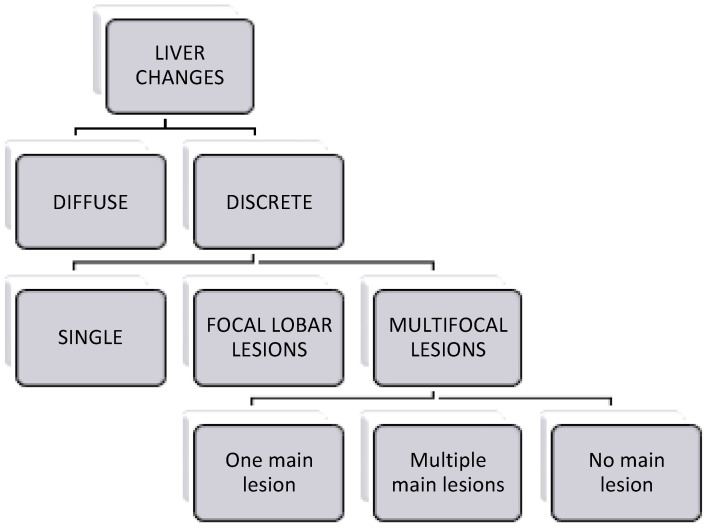
Classification of the liver changes.

The authors followed a standardised form to review the images (Supplementary Materials S1). First, the liver was classified as normal or abnormal based on overall appearance. Second, the changes were separated into two categories (Figure 1): (1) “diffuse” if the whole liver was affected diffusely, or (2) “discrete” if there was the presence of discrete lesions. If the lesion was categorised as “diffuse”, the mean attenuation of the liver (HU) and the contrast enhancement characteristics in the post-contrast studies were recorded. The discrete lesions were classified into (a) “single” (if only one lesion was present), (b) “focal lobar lesions” (if more than one lesion was present in one lobe only), and (c) “multifocal lesions” (if multiple lesions were present in more than one lobe). The “multifocal lesions” were divided into three subcategories: “One main lesion”, when a lesion more than twice the size of the others was present; “multiple main lesions”, when more than a single main lesion was present (i.e., two or more lesions more than twice the size of other lesions); and “no main lesion”, when all lesions were similar in size (Figure 1).

For the “discrete” lesions, Röentgen signs were recorded as location (liver lobe affected), size, shape, margination, and mean attention (HU). Features such as the presence of a “cluster sign” (multiple small, low-attenuation lesions aggregated into a single larger cavity), a cystic appearance (if 50% of the lesion showed attenuation equal to or below 20 HU), multilocular appearance (presence of 1 mm thick septations within the lesion), the presence of intralesional gas, the presence of a mass effect, and liver contour deformation were recorded. Post-contrast characteristics were also recorded, including the enhancing features (homogenous, heterogeneous, rim-like, or septal), the presence of transient segmental arterial enhancement, and the presence of a “double target sign”. Transient segmental arterial enhancement was defined as a transitory difference in the parenchyma surrounding an abscess, characterised by a regional or lobar hyperattenuation surrounding the abscess on the arterial phase, which becomes isoattenuating with the surrounding parenchyma on the delayed phase. A “double target sign” was defined as two concentric rings of varying attenuations surrounding a central hypoattenuating fluid-filled area, giving a stratified pattern to the lesion, resembling a “target”. Other CT findings were also recorded: the presence of peritoneal free fluid (distribution and mean attenuation), fat stranding (distribution), and pneumoperitoneum and pneumobilia. The presence of lymphadenomegaly was recorded and classified as local (when affecting the hepatic and splenic lymph nodes) and regional (when affecting the coeliac lymph centre (i.e., hepatic, splenic, gastric and pancreatico-duodenal lymph nodes). Other findings, such as the presence of thrombosis and the presence of possible infectious foci such as discospondylitis or prostatitis, were also recorded.

### 2.3. CT Acquisition Parameters

Computed Tomography studies were performed with the patient in sternal recumbency under general anaesthesia. Seven different CT scanners were used: 2-slice GE HiSpeed Dual (GE Healthcare, Buckinghamshire, UK), 4-slice GE LightSpeed Plus (GE Healthcare, Buckinghamshire, UK), 64-slice Siemens SOMATOM Perspective (Siemens Healthcare Limited, Erlangen, Germany), 64-slice GE Revolution (GE Healthcare, Buckinghamshire, UK), 320-slice CT Aquilion ONE-Genesis (Canon Medical Systems, Tokyo, Japan), 16-slice GE BrightSpeed (GE Healthcare, Buckinghamshire, UK), 16-row multi-detector helical CT Aquilion Lightning (Canon Medical Systems Corporation, Tokyo, Japan); and Hitachi Eclos 16-slice (Hitachi Healthcare, Tokyo, Japan). The CT scan parameters included helical acquisition, slice thickness ranging between 1 and 5 mm, 120 and 180 mAs, 120 and 130 kVp, a field of view 120 mm and 320 mm, matrix size 512 × 512, and a medium and a high-frequency reconstruction algorithm. Postcontrast series were performed following intravenous administration of 2 mL/kg of an iodinated non-ionic contrast agent (Omni-opaque 300 mgI/mL solution for injection, Iohexol, GE Healthcare, Oslo, Norway) using manual or automated injection.

### 2.4. Statistical Analysis

Data were stored in a Microsoft Excel (Office 365; Microsoft Corporation, Redmond, Washington, DC, USA) spreadsheet and imported into R Statistical Software (v4.3.1) [29] for all statistical analysis. The Shapiro–Wilk test was used to formally assess the normality of the distribution of dog age. Dog age (months), due to a non-normal distribution (Shapiro–Wilk *p*-value < 0.05), was described as a median with an interquartile range (IQR) and range. The remainder of the variables relating to signalment, clinical history, clinical examination, biochemistry and CBC, histopathology/cytology reports, and bacterial culture were categorical and were described as proportions (%).

The Fisher’s exact test was used to assess the relationship between bacterial shape (Cocci vs. Bacilli vs. Cocci and Bacilli) and lesion type (multifocal vs. single) as outcome variables and signalment, clinical signs, biochemistry and CBC, histopathology/cytology reports and bacterial culture.

Additionally, the relationship between lesion type and age was assessed using the Kruskal–Wallis rank sum test.

The significance threshold was set at *p* < 0.05, and significance testing was not adjusted for multiple comparisons.

## 3. Results

### 3.1. Signalment

A total of 20 dogs met the inclusion criteria. Dogs had a median age of 136.5 months (IQR 118.0, 156.0 months; range 12.0, 168.0 months). This study included nine dogs over 25 kg (45%), eight dogs between 10 kg and 25 kg (40%), and three dogs less than 10 kg (15%). Dogs included were three Border Collies (15%), five crossbreeds (25%), two Labrador Retrievers (10%), and ten dogs of other breeds (Doberman, Basset Hound, Springer Spaniel, Schnauzer, Flat-coat retriever, German Shepherd, Cocker Spaniel, Miniature Schnauzer, Nova Scotia Duck Tolling Retriever, and West Highland White Terrier) (5% each). There were 11 (55%) male neutered dogs, five (25%) female spayed dogs, three female entire dogs (15%), and one male entire dog.

### 3.2. History and Clinical Findings

Lethargy (16/20; 80%), pyrexia (>39 °C) (12/20; 60%), and anorexia or inappetence (11/20; 55%) were the most common presenting complaints, followed by other nonspecific signs, including vomiting (4/20; 20%) and weight loss (4/20; 20%).

At the time of presentation, the clinical signs had been present for two weeks or less in 16/19 cases (84.2%) and over two weeks in 3/19 cases (15.8%). During the clinical exam, ten of 19 (52.6%) dogs showed abdominal pain, 10 (52.6%) pyrexia, 7 (36.8%) tachycardia (defined as a heart rate > 120/min), and 2 (10.5%) tachypnoea (defined as a breathing rate > 30/bpm). The length of clinical signs and physical examination were unknown in one of the 20 cases. A total of 12 of 18 dogs (66.7%) were alert, and six (33.3%) were depressed. Information regarding demeanour was not available in two of the 20 cases.

### 3.3. Laboratory Findings

A complete blood count was performed in 19 of 20 cases, with anaemia noted in eight (42.1%) and neutrophilia in 13 (68.4%). Results of serum biochemical analysis were available in 19 of 20 dogs, with alanine aminotransferase (ALT) raised in 17 (89.5%) and alkaline phosphatase (ALKP) in 13 cases (68.4%).

### 3.4. Cytological, Histological and Bacteriological Findings

Histopathology of lesions resected during surgery or examined at necropsy was performed in seven of 20 cases (38.9%), with neutrophilic hepatitis and intralesional bacteria observed in all of them (100%). The bacteria observed were Bacilli in two (28.6%) of the samples, Cocci in three (42.9%), a combination of Cocci and Bacilli in one, and a weakly Gram-positive and non-acid-fast filamentous bacteria in another case. There was no evidence of neoplastic cells in histopathology in any of the samples. None of the cases fell into the category of ‘’histology without evidence of bacteria and positive bacterial culture” OR “histology without evidence of bacteria and a positive FISH (fluorescence in situ hybridisation) or PCR for bacteria”.

Ultrasound-guided percutaneous fine needle aspiration of hepatic lesions was performed in 16 of 20 cases (80%), with all of them (100%) revealing bacteria upon cytological examination. Fifteen of these 16 (94.5%) cases described neutrophilic inflammation within intracellular bacteria, and a single case showed histiocytic/macrophagic inflammation with intracellular bacteria. The shape of the bacteria observed was Cocci in nine (60%) of the samples, Bacilli in three (20%), and a combination of Cocci and Bacilli in three (20%). There was no evidence of neoplastic cells in any of the samples. None of the cases fell into the category “cytology without evidence of bacteria and positive bacterial culture”.

Bacteria were isolated from the main liver lesions in 12 of 20 cases (60%), with one of these cases growing >1 organism. The organisms isolated were *E. coli* (4/12, 33.3%), *Streptococcus* spp. (3/12, 24.9%), *Staphylococcus* spp. (2/12, 16.6%), *Salmonella* spp. (2/12, 16.6%), *Clostridium perfringens* (1/12), and *Enterococcus* spp. (1/12).

### 3.5. CT Studies

Sixteen CT studies (80%) included the thorax and abdomen, and four (20%) included the abdomen only. All studies included pre-contrast series, nine studies included venous post-contrast phase only (45%), eight studies included arterial and venous phase (40%), two studies included arterial, portal, and venous phases (10%), and one study included portal and venous phases.

### 3.6. CT Findings

The liver was deemed abnormal in all cases. Twelve livers were described as normal size (60%), seven were enlarged (35%), and one was small. The liver changes were described as “discrete” in 19 cases (95%) and “diffuse” in 1 case (5%).

#### 3.6.1. “Diffuse” Changes

**Figure 2:**
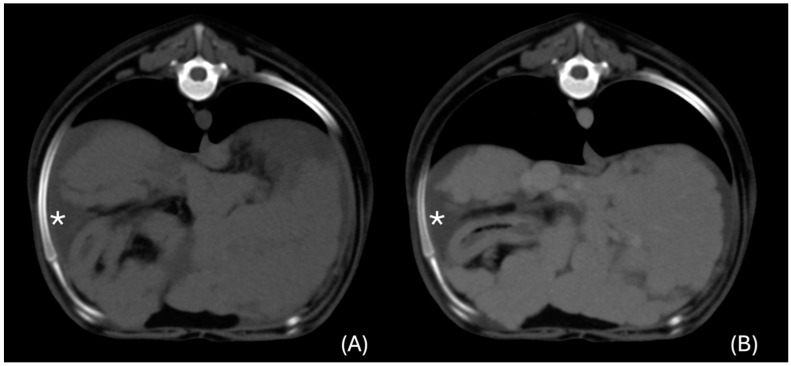
Transverse unenhanced (**A**) and venous phase (**B**) images at the cranial abdomen (window level 50 HU, window width 350 HU). Diffuse liver changes were present, characterised by irregular margination of the liver lobes and homogeneous contrast enhancement. Peritoneal effusion was present (*).

In one case, the abnormalities involved homogeneously the entire liver. The liver was increased in size, with an irregular and nodular contour. The pre-contrast attenuation was 65 HU, with a homogenous and mild post-contrast enhancement in the portal (73 HU) and venous (100 HU) phases (Figure 2).

#### 3.6.2. “Discrete” Changes

Of the “discrete” cases, the lesions were described as multifocal in 14 cases (73.7%) and single in five cases (26.3%). No lesions were described as focal lobar lesions. Of the multifocal lesions, “one main lesion” was reported in 11 cases (57.9%), “no main lesion” was reported in 2 cases (10.5%), and “multiple main lesions” were reported in one case.

For the location of the main liver lesions, the two livers classified as multifocal with the subdivision of “no main lesions” were not counted, as a main lesion was not identifiable. Of the remaining livers (17), 18 main lesions were present, as there was a liver with 2 main lesions. A total of 11 of the 18 lesions were present in the left hepatic division, and 7 were located on the right. Seven lesions were present on the left lateral liver lobe, four on the left medial, four on the right lateral, and three on the right medial one.

**Figure 3:**
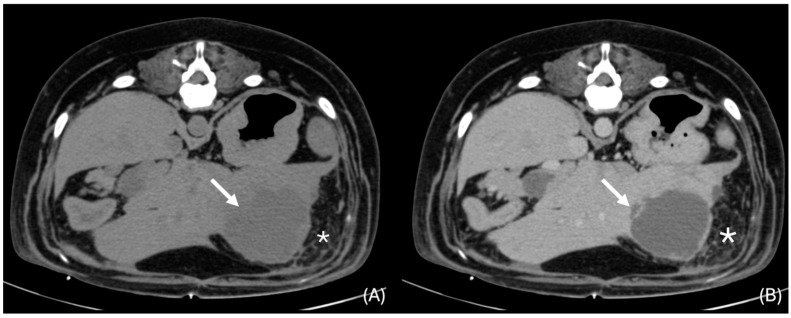
Transverse unenhanced (**A**) and venous phase (**B**) images of a hypoattenuating lesion that deforms the liver contour (white arrows) in the left lateral liver lobe (window level 50 HU, widow width 350 HU). Note the presence of adjacent focal peritoneal fat stranding (*).

All the livers showed attenuation in the normal reported range (60–70 HU) [30], and, in all the cases but one, the lesions were of lower attenuation to that of the normal liver (ranging between 15 to 53 HU (Figure 3). The lesions were cystic in 5 cases (26.3%) and soft tissue attenuating (ranging between 25 and 53 HU) in the remaining 14 cases (73.7%). All lesions were ovoid to rounded in appearance. The lesions caused a mass effect in 11 cases (57.9%) and deformed the liver contour in 14 cases (73.7%). Liver lesions were visible in the pre-contrast and post-contrast series in 18 cases (94.7%), and in 1 case, the lesion was visible only in the post-contrast portal phase. The lesions were described as well-defined in the post-contrast series in 16 cases (84.2%) and ill-defined in three cases (15.8%). In the pre-contrast studies, nine cases were described as well-defined (47.4%) and ten as ill-defined (52.6%).

**Figure 4:**
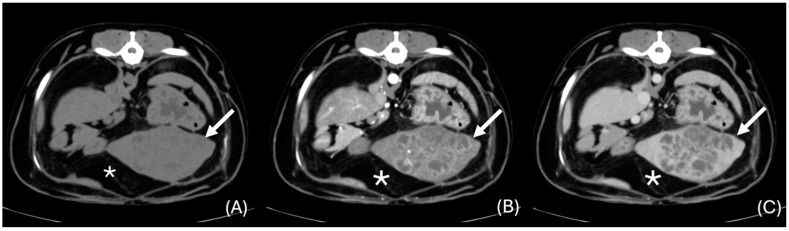
Transverse unenhanced (**A**), arterial phase (**B**), and venous phase (**C**) images of a large lesion showing the “cluster sign” (white arrows) and septal enhancement in the left lateral liver lobe (window level 50 HU, widow width 350 HU). Note the presence of adjacent focal peritoneal fat stranding (*).

**Figure 5:**
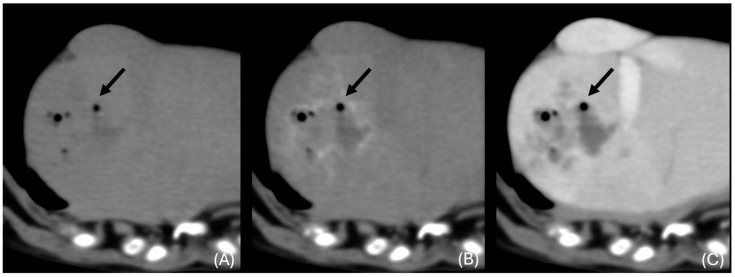
Transverse unenhanced (**A**), arterial phase (**B**), and venous phase (**C**) close-up images at the level of the right medial liver lobe (window level 50 HU, widow width 350 HU). A cluster sign, rim enhancement in the arterial phase, and intralesional gas were noted (black arrows).

**Figure 6:**
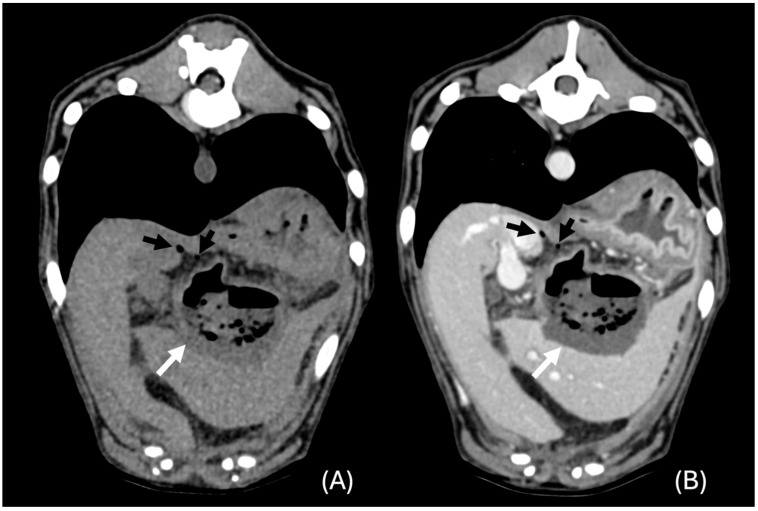
Transverse unenhanced (**A**) and portal phase (**B**) images at the left lateral liver lobe (window level 50 HU, widow width 350 HU). A hypoattenuating lesion with intralesional gas was present (thick white arrows). Note also the presence of pneumoperitoneum (black arrows).

**Figure 7:**
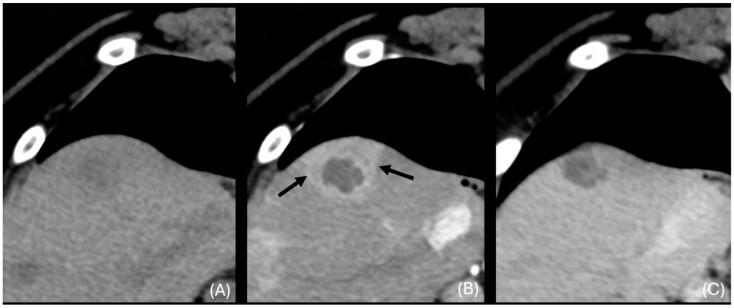
Transverse unenhanced (**A**), arterial phase (**B**), and venous phase (**C**) images at the right medial liver lobe (window level 50 HU, widow width 350 HU). Transient segmental arterial enhancement is present (black arrows).

A cluster sign was observed in eight cases (42.1%) (Figure 4 and Figure 5). The lesions were multilocular in 11 cases (61.1%) and unilocular in seven (38.9%). Gas was present within the lesions in four cases (20%) (Figure 5 and Figure 6). Transient segmental arterial enhancement was present in six of the 10 cases in which an arterial phase was available (60%) (Figure 7). Rim-like enhancement (in either arterial, portal, or venous phases) was noted in 6 cases (31.6%) (Figure 5), and septal enhancement was present in 11 cases (57.9%) (Figure 4).

### 3.7. Additional Changes Accompanying the Presence of Liver Infection

Regarding additional changes in CT, fat stranding was present in 14 cases (70%) (Figure 3), and peritoneal free fluid was present in 13 cases (65%) (Figure 2). Lymphadenomegaly was the most common finding, present in 18 cases (90%). In those cases, lymphadenomegaly was present limited to the local lymph nodes in 11 cases (61.1%) and involved the regional lymph nodes in seven cases (38.9%). In five cases, other lymph nodes were also affected: four cases of jejunal lymphadenomegaly and one case of medial iliac lymphadenomegaly.

Pneumoperitoneum was present in four cases (20%) (Figure 5), but one of the cases had a recent exploratory laparotomy 24 h prior to referral, and another case had surgery to obtain falciform fat for stem cell therapy 14 days prior to referral. Portal vein thrombosis was noted in one case, located at the left branch of the hepatic portal vein. The biliary system showed abnormalities in eight cases (40%): pneumobilia (three cases), presence of cholecystoliths (five cases), and distension of the common bile duct (three cases).

Other abnormalities noted were pleural effusion (four cases), sternal lymphadenomegaly (seven cases), and one case of endplate lysis suggestive of discospondylitis.

### 3.8. Association of CT Findings with Cytology/Histopathology/Culture

Bacterial shape was not found to be associated with any of the variables tested (Table 1).

Lesion type was only associated with the presence of abdominal pain (*p* = 0.023), with abdominal pain being present in 55.6% of cases with multifocal lesions but zero cases with single lesions (Table 2). Lesion type was additionally not associated with the dog’s age (*p* = 0.643).

## 4. Discussion

The aim of this study was to characterise the CT findings of liver bacterial infections in dogs and compare them to those described in the human literature, contributing to the understanding of this pathology. The CT findings in all the cases with hepatic discrete lesions in this study were compatible with the reported features of human pyogenic abscesses. These lesions were primarily multifocal discrete lesions, rounded to ovoid, and of variable size. Except for one case, all the discrete lesions were hypoattenuating compared to the surrounding liver parenchyma, and all were hypoattenuating in post-contrast series against the normally enhancing liver. Hypoattenuating liver lesions are also a typical CT characteristic of hepatic abscessation in dogs, as previously reported [5,20,25]. Pyogenic liver abscesses in humans are typically hypoattenuating lesions with various features, such as non-localised fluid collection, multiloculate cystic mass, or multifocal lesions [3,8,9,10]. These different CT appearances can be partially explained by variable aetiologies, with multifocal liver abscessation commonly associated with biliary origin, multifocal micro-abscessation (<2 cm diameter) associated with enteric organisms or haematogenous spread, and single hepatic lesions being usually cryptogenic in origin [12,31].

Less than half of the discrete lesions in this study were well-defined on pre-contrast images compared to most being well-marginated post-contrast. This feature has also been reported in humans [8] and should prompt clinicians to perform post-contrast images for better delineation of these lesions.

Most discrete lesions were located at the left hepatic division, particularly the left lateral liver lobe. This is contrary to humans, where the right lateral liver lobe is usually the most affected, probably due to the larger proportion of portal blood this lobe receives [32]. We could theorise that this difference may be explained by the larger size of the left portal branch in dogs and the subsequent larger size of the left hepatic division, making this part of the liver more prone to infections ascending from the portal system [33,34].

Rim-like enhancement was seen in about a third of our cases with discrete lesions. This CT feature has been described in up to 38% of human liver abscesses in CT [14] and has also been reported in three hepatic abscesses in dogs [25]. Rim enhancement represents an inflammatory and pyogenic membrane in the abscesses [15], and it has also been described in bacterial abscesses in the musculature in dogs [35]. However, this CT feature is nonspecific, as has also been described in other neoplastic and non-neoplastic liver diseases [36,37,38].

Almost half of the cases of discrete lesions in our study show the characteristic cluster sign described in human literature, with approximately 30% to 80% of cases with pyogenic liver abscesses showing this CT feature in humans [13], and it is also associated with abscesses of biliary or enteric origin [12,39]. These imaging changes represent an early or immature stage of the abscess evolution [10], which may explain why it is not visible in a higher number of cases, as it has been theorised that these abscesses could coalesce in a larger multiseptated lesion or a homogeneously unilocular and fluid-filled abscess in a later and more advanced stage of the disease [13]. Although not pathognomonic, as it was described in a case of metastatic carcinoma in humans [13], we considered that the “cluster sign” should be deemed highly suggestive of pyogenic liver abscess in dogs.

Transient segmental arterial enhancement was seen in most cases with discrete lesions when an arterial phase was available. This imaging finding is similar to the human literature, with up to 67% of abscesses showing this CT sign [14]. This appearance is due to the obstruction and consequent reduction in the portal flow with a compensatory increase in the arterial flow [14]. Segmental enhancement can also be found in neoplastic or arterioportal shunts, although the wedge shape is more characteristic of pyogenic liver abscesses [14].

Despite being one of the most typical CT findings in pyogenic liver abscess in humans [15], the “target-like” sign was not present in our study in any of the cases. An outer hypoattenuating halo was not observed in our cases, which in humans represents parenchymal oedema [15]. We could speculate that the pathophysiology of pyogenic liver abscesses in dogs and humans differs or that the liver abscesses in our study were in a different state of maturity in which this oedematous halo is absent. The maturation process of a pyogenic abscess includes the angiogenesis of permeable vessels in the capsule, through which inflammatory cells enter the infected area. Subsequently, the abscess core liquefies as the inflammatory cells clear the infection, and the abscess consolidates and retracts [40]. Considering that oedema is associated with acute states of inflammation [41], it is possible that our cases did not show this oedematous halo if they were not in the acute or early stages of the disease process.

Gas was visible within less than a fourth of our discrete lesions (20%). Emphysematous liver abscesses have been reported in humans, present in approximately 20% of pyogenic liver abscesses [3]. In dogs, approximately 23% of cases show this feature in ultrasonographic studies [17]. This feature is caused by gas-forming bacteria such as *E. coli*, *Clostridium* spp., or *Streptococcus* spp. [42]. However, gas-containing lesions in the liver are not exclusive of the pyogenic liver abscesses and are also associated with necrotic processes such as liver neoplasia in humans and dogs [43,44] and lobar torsions in dogs [21]. Gas within the liver parenchyma has also been reported as emphysematous hepatitis in both species [45]; however, the presence of additional features such as a wall, cluster sign, and multiseptated appearance in CT prioritise a liver abscess as a main differential in humans [46,47]. The authors suggest that although gas within hepatic lesions has been historically associated with pyogenic liver abscesses in veterinary medicine, this feature should be considered carefully and interpreted with the presence of other CT features.

Most of our cases presented additional imaging findings of local or regional lymphadenopathy, peritoneal free fluid, and peritoneal fat stranding. This has also been previously reported in bacterial hepatic liver abscesses in dogs [16,17], and it is expected due to the inflammatory character of this entity [48].

The biliary system showed abnormalities in eight cases, including pneumobilia, cholelithiasis, and distension of the common bile duct. These abnormalities have been previously associated with infectious processes of the biliary tree in dogs but also with non-infectious diseases or even observed in clinically normal dogs [49,50,51]. Biliary tract infection has been suggested as a potential cause of human bacterial liver abscessation [5]. The significance of these findings in our cases is unknown, particularly in the absence of bile culture, which has been used as a gold standard for diagnosing hepatobiliary infection in small animals [52].

Our case with diffuse hepatic changes was also the only one showing histiocytic/macrophagic inflammatory changes in cytology suggestive of granulomatous hepatitis and yielding acid–alcohol-resistant bacillary bacteria. This patient was a Schnauzer, and this breed has been reported to have a predilection for *Mycobacterium avium complex* (MAC), an acid–alcohol-resistant bacteria, due to a mutation in a specific gene (CARD9) [53,54]. Our patient was tested and proved homozygous for the mutation in this gene, so a presumptive diagnosis of *M. avium* complex infection was reached. There is a limited description of the imaging features of tuberculosis infection in dogs, with a single case reporting a CT finding of multiple rounded, well-defined, non-contract-enhancing nodules of 30 HU scattered through the liver that yielded *Mycobacterium tuberculosis* [28]. Our case showed hepatomegaly, with an irregular and nodular contour and homogenous contrast enhancement. According to our review of the literature, this is the first CT description of an *M. avium* liver infection in a dog. However, due to the irregular contour of the liver in this dog, concomitant chronic hepatitis could not be excluded entirely, although this condition usually comes with a small liver size in its advanced stages [55].

Our study did not retrieve any cases of acute bacterial hepatitis. We could speculate that due to the nonspecific imaging findings in cases of acute hepatitis, clinicians usually opt not to sample these livers. In addition, *Leptospira* spp., the most recognised infectious cause of acute hepatitis in dogs and also associated with chronic granulomatous hepatitis [56], is challenging to diagnose in liver samples, requiring advanced laboratory tests such as PCR, silver staining of biopsy, immunohistochemistry, or in situ hybridisation [57]; this could have reduced the possibility of retrieving cases of Leptospirosis for our study.

Acute history of lethargy, pyrexia, anorexia/inappetence, and vomiting were the most common clinical presentations in our cases, with abdominal pain, hyperthermia, and tachycardia seen as the most typical findings in the clinical exam. The most common laboratory findings were anaemia, neutrophilia, and raised liver enzymes (alanine transaminase and alkaline phosphatase). These clinic–pathologic data are similar to previous studies of liver abscessation and liver infection in dogs, being nonspecific and most often consistent with hepatobiliary disease, making the diagnosis of these pathologies challenging [5,17,20].

Neutrophilic inflammation and intralesional bacteria were diagnosed in all liver samples with discrete lesions, whether in cytology or histopathology, which are typical cytological findings of pyogenic liver abscesses [58]. Cocci were the most common bacterial shape, followed by Bacilli and a combination of both bacteria in cytology and histopathology. *E. coli* was the most common microorganism cultured, similar to previous reports of liver abscesses in dogs and in humans [3,5,17,20], and is associated with biliary or enteric infections [31]. Polymicrobial infections have been previously reported in pyogenic bacterial liver abscesses in dogs [5,17], and they count for the majority of infection in liver abscesses in cats and humans [31,59], being also related to biliary or gastrointestinal origin with a predominance of Gram-negative and anaerobic bacteria in humans [31]. Only one of our cases yielded more than one bacterium in culture; however, anaerobic organisms are difficult to isolate and may require special laboratory techniques, so polymicrobial infections were likely underestimated in our study, as previously occurred in other studies [5].

There are some limitations of this study, which are related to the design. We lack positive culture in 40% of our cases, and this technique represents the gold standard for diagnosing pyogenic liver abscesses in humans [11]. However, the resemblance of these lesions to human pyogenic liver abscesses in CT and the direct visualisation of bacteria within the liver lesions allowed us to classify our cases as presumed canine pyogenic liver abscesses. In addition, a negative culture can be found in up to 20% of pyogenic liver abscesses in humans due to poor culture techniques or the use of broad-spectrum antibiotics [12]. Another limitation is related to the correlation between lesion sampling and cytology/culture results. Given that in most of our cases, more than one lesion was observed in the liver, it is not possible to know precisely which lesion was sampled. It was assumed that the largest or most accessible lesions would have been sampled, and the results were assumed to represent the entire liver disease process. This limitation can be found in other studies with multiple lesions in CT or ultrasound, in which the sampling of every lesion seen in the studies is not specified [17,60,61]. We acknowledge that we cannot know for sure which lesion corresponds to pyogenic liver abscesses, as post-mortem histopathology would be required for every case. The lack of cases affected by Leptospira spp. is another limitation, considering the high prevalence of this bacteria in the regions where our cases were evaluated [62,63]. As explained earlier in the discussion, it is likely that given the difficulty of diagnosing leptospirosis in liver samples, these cases were not included in this study due to the inclusion criteria. In addition, the gold standard for diagnosing leptospirosis in dogs is the Microscopic Agglutination Test (MAT) [64], which is performed in blood samples.

Another limitation related to the sampling is the assumption of primary liver infection with no neoplastic process associated. The majority of our samples were cytologically examined, and even though fine needle aspiration has been showing high sensitivity for diagnosing liver lesions in humans, the sensitivity diagnosing benign liver tumours has been proven low (40%) [65,66], so the presence of benign neoplasia such as liver adenomas in our cases cannot be completely ruled out.

The small sample size is a significant limitation and prevented the statistical analysis of other changes, for example, the association between the CT appearance of the lesions and the type of bacteria or clinicopathological findings. An association was found between multifocal liver lesions and patients showing abdominal pain, despite the small sample size. We can theorise that the involvement of different liver lobes could trigger pain in a more extensive area of the abdomen during palpation compared to when a single lobe is affected. The small number of cases also prevented the statistical analysis of the different acquisition parameters and the value of the post-contrast sequences. Further studies with a larger number of cases would be required to investigate this further.

Variable CT protocols, due to the multicentric nature of the study, made classification of post-contrast abnormalities challenging and further reduced the sample size to interpret abnormalities such as the transient segmental arterial enhancement and perform statistical analysis. Prospective studies with standardised protocols and greater case numbers could be conducted to evaluate these canine liver abscessation features.

## 5. Conclusions

In conclusion, the CT characteristics of pyogenic liver abscesses in our study resemble those described in the human literature, with multifocal or single, round or ovoid, hypoattenuating liver lesions, which are better-visualised post-contrast images and can show features such as the “cluster sign”, transient segmental arterial enhancement, rim enhancement, and intralesional gas. Additional CT findings, such as local lymphadenomegaly, peritoneal fat stranding, and peritoneal free fluid, were also commonly observed in this study.

## Figures and Tables

**Table 1:** The relationship between bacteria shape and histopathology/cytology reports in 20 dogs with liver parenchymal infections.

	Bacterial Shape			Fisher’s Exact Test *p*-Value
	Bacilli	Cocci	Cocci and Bacilli	
*Gas visible*				0.591
Yes	2 (10.0%)	2 (10.0%)	0 (0.0%)	
No	4 (20.0%)	9 (45.0%)	3 (15.0%)	
*Cluster sign*				1.00
Yes	2 (10.5%)	5 (26.3%)	1 (5.3%)	
No	3 (15.8%)	6 (31.6%)	2 (10.5%)	
*Cystic appearance*				0.787
Yes	1 (5.3%)	4 (21.1%)	0 (0.0%)	
No	4 (21.1%)	7 (36.8%)	3 (15.8%)	
*Peritoneal free fluid*				0.574
Yes	4 (20.0%)	8 (40.0%)	1 (5.5%)	
No	2 (10.0%)	3 (15.0%)	2 (10.0%)	
*Fat stranding*				0.357
Yes	5 (25.0%)	8 (40.0%)	1 (5.0%)	
No	1 (5.0%)	3 (15.0%)	2 (10.0%)	
*Pneumoperitoneum*				1.00
Yes	1 (5.0%)	3 (15.0%)	0 (0.0%)	
No	5 (25.0%)	8 (40.0%)	3 (15.0%)	
*Lymphadenomegaly*				0.363
Yes	6 (30.0%)	10 (50.0%)	5 (25.0%)	
No	0 (0.0%)	1 (5.0%)	1 (5.0%)	

**Table 2:** The relationship between lesion type and signalment, clinical signs, biochemistry and CBC, histopathology/cytology reports, and bacterial culture in 19 dogs with hepatic changes due to parenchymal liver infection.

	Lesion Type		Fisher’s Exact Test *p*-Value
	Multifocal	Single	
*Body weight category*			1.00
<10 kg	2 (11.1%)	0 (0.0%)	
10–25 kg	5 (27.8%)	2 (11.1%)	
>25 kg	7 (38.9%)	2 (11.1%)	
*Sex*			1.00
Female	6 (31.6%)	2 (10.5%)	
Male	8 (42.1%)	3 (15.8%)	
*Neuter status*			1.00
Neutered	11 (57.9%)	4 (21.1%)	
Entire	3 (15.8%)	1 (5.3%)	
*Anorexia/inappetence*			0.338
Yes	7 (36.8%)	4 (21.1%)	
No	7 (36.8%)	1 (5.3%)	
*Pyrexia*			0.305
Yes	10 (52.6%)	2 (10.5%)	
No	4 (21.1%)	3 (15.8%)	
*Vomiting*			0.272
Yes	2 (10.5%)	2 (10.5%)	
No	12 (63.2%)	3 (15.8%)	
*Weight loss*			0.272
Yes	2 (10.5%)	2 (10.5%)	
No	12 (63.2%)	3 (15.8%)	
*Clinical presentation*			0.405
Acute (<2 weeks)	13 (72.2%)	3 (16.7%)	
Chronic (>2 weeks)	1 (5.6%)	1 (5.6%)	
*Previous antibiotic treatment*			1.00
Yes	5 (27.8%)	1 (5.6%)	
No	9 (50.0%)	3 (16.7%)	
*Demeanour*			0.237
Alert	7 (41.2%)	4 (23.5%)	
Depressed	6 (35.3%)	0 (0.0%)	
*Abdominal pain*			0.023
Yes	10 (55.6%)	0 (0.0%)	
No	4 (22.2%)	4 (22.2%)	
*Tachycardia*			1.00
Yes	5 (27.8%)	2 (11.1%)	
No	9 (50.0%)	2 (11.1%)	
*Tachypnoea*			0.405
Yes	1 (5.6%)	1 (5.6%)	
No	13 (72.2%)	3 (16.7%)	
*Anaemia*			1.00
Yes	6 (33.3%)	1 (5.6%)	
No	8 (44.4%)	3 (16.7%)	
*Neutrophilia*			0.533
Yes	11 (61.1%)	2 (11.1%)	
No	3 (16.7%)	2 (11.1%)	
*Raised alanine aminotransferase (ALT)*			1.00
Yes	12 (66.7%)	4 (22.2%)	
No	2 (11.1%)	0 (0.0%)	
*Raised alkaline phosphatase (ALKP)*			0.533
Yes	11 (61.1%)	2 (11.1%)	
No	3 (16.7%)	2 (11.1%)	
*Bacteria shape*			0.276
Bacilli	4 (21.1%)	1 (5.3%)	
Cocci	9 (47.4%)	2 (10.5%)	
Bacilli and Cocci	1 (5.3%)	2 (10.5%)	
*Liver size*			0.081
Decreased	0 (0.0%)	1 (5.6%)	
Normal	8 (42.1%)	4 (21.1%)	
Increased	6 (31.6%)	0 (0.0%)	
*Peritoneal free fluid*			1.00
Yes	9 (47.4%)	3 (15.8%)	
No	5 (26.3%)	2 (10.5%)	
*Fat stranding*			1.00
Yes	10 (52.6%)	3 (15.8%)	
No	4 (21.1%)	2 (10.5%)	
*Pneumoperitoneum*			1.00
Yes	3 (15.8%)	1 (5.6%)	
No	11 (61.1%)	4 (21.1%)	
*Lymphadenomegaly*			0.468
Yes	13 (68.4%)	4 (21.1%)	
No	1 (5.6%)	1 (5.6%)	

## Data Availability

The data presented in this study are not publicly available due to privacy and security protection. An Excel spreadsheet is available from the authors upon request.

## Supplementary Materials

The following supporting information can be downloaded at: https://www.mdpi.com/article/10.3390/ani14233399/s1, Standardised form to review the images.
